# Comparing the risk of cardiovascular disease between degarelix and gonadotropin-releasing hormone agonists:a systematic review and meta-analysis

**DOI:** 10.3389/fonc.2025.1523794

**Published:** 2025-10-01

**Authors:** Wencong Liu, Zhenyu Liu, Liangdong Song, Huixuan Zhu, Yu Luo, Jindong Zhang, Shuai Su, Delin Wang

**Affiliations:** ^1^ Department of Urology, The First Affiliated Hospital of Chongqing Medical University, Chongqing, China; ^2^ Department of Urology, Urologic Surgery Center, Xinqiao Hospital, Third Military Medical University (Army Medical University), Chongqing, China

**Keywords:** prostate cancer, degarelix, GnRH agonists, androgen deprivation therapy, meta-analysis

## Abstract

**Background:**

Regarding the comparison of cardiovascular disease risk between gonadotropin-releasing hormone (GnRH) antagonists and GnRH agonists, there are discrepancies in results from different studies. Therefore, this meta-analysis was conducted to investigate whether degarelix could reduce cardiovascular disease risk.

**Methods:**

We systematically searched the PubMed, Embase, Web of Science, and Cochrane Library databases with a search time limit of up to December 2023 for articles focusing on the use of degarelix, a GnRH antagonist, in prostate cancer, with an emphasis on articles comparing degarelix to GnRH agonists. Study endpoints included major adverse cardiovascular events, stroke, all-cause mortality, myocardial infarction, heart failure, and arrhythmia.

**Results:**

A total of 1320 articles were retrieved, of which eight met our inclusion criteria and involved 138–065 patients. The pooled results showed no difference in the risk of major adverse cardiovascular events (hazard ratio [HR]=0.94, 95% confidence interval [CI]: 0.65–1.35; P=0.73), stroke (HR=0.89, 95% CI: 0.62–1.27; P=0.52), myocardial infarction (HR=0.98, 95% CI: 0.70–1.37; P=0.91), all-cause mortality (HR=1.09, 95% CI: 0.73–1.65; P=0.67), and arrhythmia (risk ratio=0.64, 95% CI: 0.15–2.76; P=0.55) between degarelix and GnRH agonists. However, degarelix reduced the risk of heart failure (HR=0.56, 95% CI: 0.36–0.88; P=0.01).

**Conclusion:**

Further clarification on the effects of different androgen deprivation therapy modalities on cardiovascular disease is needed from future and larger prospective randomized controlled trials.

## 1 Introduction

With the exception of non-melanoma skin cancer, prostate cancer is the most common type of cancer diagnosed in males and the second largest cause of cancer-related deaths in the United States (US) (1). The incidence of prostate cancer was estimated to increase by 2–3% per year between 2015 and 2019; thus, the number of newly diagnosed prostate cancer cases in the US in 2024 is estimated to exceed 290 000, and the number of predicted deaths is estimated to exceed 35 000 (2).

The development of prostate cancer depends on androgens and androgen receptors; therefore, androgen deprivation therapy (ADT) is a commonly used treatment for the disease (3). ADT can be categorized into two main groups: drug treatment and surgical castration. Surgical castration is an orchiectomy, and the drugs used for therapy include gonadotropin-releasing hormone (GnRH) agonists and GnRH antagonists (4, 5). Owing to the irreversibility of orchiectomy and its psychological impact on patients, it is gradually being replaced with drug therapy. Currently, the commonly used GnRH agonists include leuprorelin, goserelin, buserelin, and triptorelin, whereas GnRH antagonists include degarelix and relugolix, the former being administered via subcutaneous injection and the latter administered orally (6). Some studies have suggested that ADT increases the risk of cardiovascular disease (7, 8), which is the most common cause of death in patients with prostate cancer (9).

Degarelix inhibits the excitatory effects of endogenous GnRH on the pituitary gland by competitively binding to GnRH receptors, thereby inhibiting follicle-stimulating hormone (FSH) and luteinizing hormone (LH) production and directly decreasing testosterone levels such that no testosterone surge occurs. Results from a 1-year, randomized, open-label phase III trial (CS21) showed that degarelix was similar to leuprorelin in inducing and maintaining low serum testosterone levels (≤0.5 ng/mL); it significantly induced prostate-specific antigen and testosterone suppression faster than leuprorelin (10). GnRH agonists, however, regulate testosterone levels through a negative feedback pathway mechanism of the hypothalamic-pituitary-gonadal axis; the initial use of the drug can lead to a sharp increase in testosterone levels, and the increase in testosterone may induce or exacerbate urinary retention, bone pain, and spinal cord compression, leading to worsening of clinical symptoms (6, 11, 12). It has been suggested that the transient increase in testosterone induced by GnRH agonists promotes angiogenesis and neutrophil aggregation in atherosclerotic plaques, leading to plaque instability and an increased likelihood of rupture (13), which may be one of the reasons why GnRH agonists are associated with a greater risk of cardiovascular disease than GnRH antagonists. Additionally, it has been proposed that GnRH antagonists inhibit both LH and FSH, whereas GnRH agonists primarily inhibit LH, and that the difference in FSH between the two may explain the difference in cardiovascular disease risk (5, 14). Although GnRH agonists cause testosterone levels to fluctuate, both GnRH agonists and antagonists suppress serum testosterone levels, which are independent predictors of metabolic syndrome in men (15, 16), and increase the risk of cardiovascular disease.

The main mechanisms of using GnRH agonists in clinical practice include the initial “Flare-up effect” and long-term effects (continuous excitation leads to pituitary desensitization and eventually inhibits testosterone to castration levels (<50 ng/dL)). The main mechanisms of GnRH antagonists include direct receptor blocking, rapid testosterone reduction (to castration levels within 24 hours), and sustained inhibition. The advantages of GnRH antagonists in cardiovascular integrity have been supported by some studies, and they are suitable for patients with concurrent cardiovascular diseases or those requiring rapid testosterone suppression. However, GnRH agonists remain the standard choice for most patients in the stable stage due to their relatively low cost. Clinical decisions need to take into account the disease stage, complications, economic factors and patient preferences comprehensively, and be dynamically adjusted with reference to the latest guidelines. There is still controversy regarding the risk of cardiovascular disease between GnRH antagonists and agonists, with some studies suggesting similar risk (17–19), and others suggesting that GnRH antagonists reduce the risk of cardiovascular disease (20–25). Owing to this, we conducted a review and meta-analysis of published results to explore whether degarelix, a GnRH antagonist, reduces the risk of cardiovascular disease.

## 2 Materials and methods

### 2.1 Search strategy

We conducted and report this meta-analysis in accordance with the Preferred Reporting Items for Systematic Reviews and Meta-Analyses statement and registered it with the International Prospective Register of Systematic Reviews (ID: CRD42024503998). We systematically searched the PubMed, Embase, Web of Science, and Cochrane Library databases with a search time limit up to December 2023 for articles focusing on the use of degarelix in patients with prostate cancer. We searched the following combination of Medical Subject Headings (MeSH) and related keywords: ‘Prostatic Neoplasms [Mesh] or Prostate Neoplasms or Prostate Cancer or Prostatic Cancer’ and ‘degarelix’.

### 2.2 Inclusion and exclusion criteria

We developed inclusion criteria on the basis of the PICOS principles: (1) population, patients diagnosed with prostate cancer by histopathologic examination; (2) intervention, treatment of prostate cancer with degarelix; (3) comparison, treatment of prostate cancer with GnRH agonists; (4) outcome, comparison of the risk of cardiovascular disease between degarelix and GnRH agonists, including major adverse cardiovascular events (MACEs, defined as the composite endpoint of stroke, myocardial infarction, or death from any cause), stroke, all-cause mortality, myocardial infarction, heart failure, and arrhythmia; and (5) study design, we had no restrictions on the article study design. The exclusion criteria were as follows: lack of relevant outcome indicators, studies that did not discuss cardiovascular disease risk, reviews, commentaries, letters, conference abstracts, and animal studies.

### 2.3 Quality assessment and data extraction

Two independent researchers reviewed the titles and abstracts of the studies. Then, a full-text search of articles meeting the inclusion criteria was performed, and quality assessment and data extraction were completed. In cases of disagreement, a decision was made after discussion with a third researcher. Two independent researchers extracted the following data from the articles based on a pre-designed table: authors, date of publication, country, study design, sample size, and treatment. For randomized controlled trials, the Risk of Bias tool (RoB 2) was used for quality assessment, while the Newcastle–Ottawa Scale (NOS) was used for the quality assessment of non-randomized controlled trials. Disagreements between the researchers were resolved through negotiation.

### 2.4 Statistical analysis

Study effect indicators are presented as hazard ratios (HRs) and corresponding 95% confidence intervals (CIs) or relative risks (RRs) and corresponding 95% CIs. For our meta-analysis, we calculated the overall HR or RR and 95% CI using Stata (version 15.0; StataCorp, College Station, TX, USA). The I^2^ test was used to assess heterogeneity across studies, using a random-effects model if I^2^ > 50% and a fixed-effects model if I^2^ < 50%. If heterogeneity was evident, a subgroup analysis was performed to determine the source. We used the Egger test to assess publication bias, which suggested the presence of publication bias if the P-value was <0.05. We also performed sensitivity analysis using the literature-by-exclusion method to assess the robustness of the results.

## 3 Results

**Figure 1:**
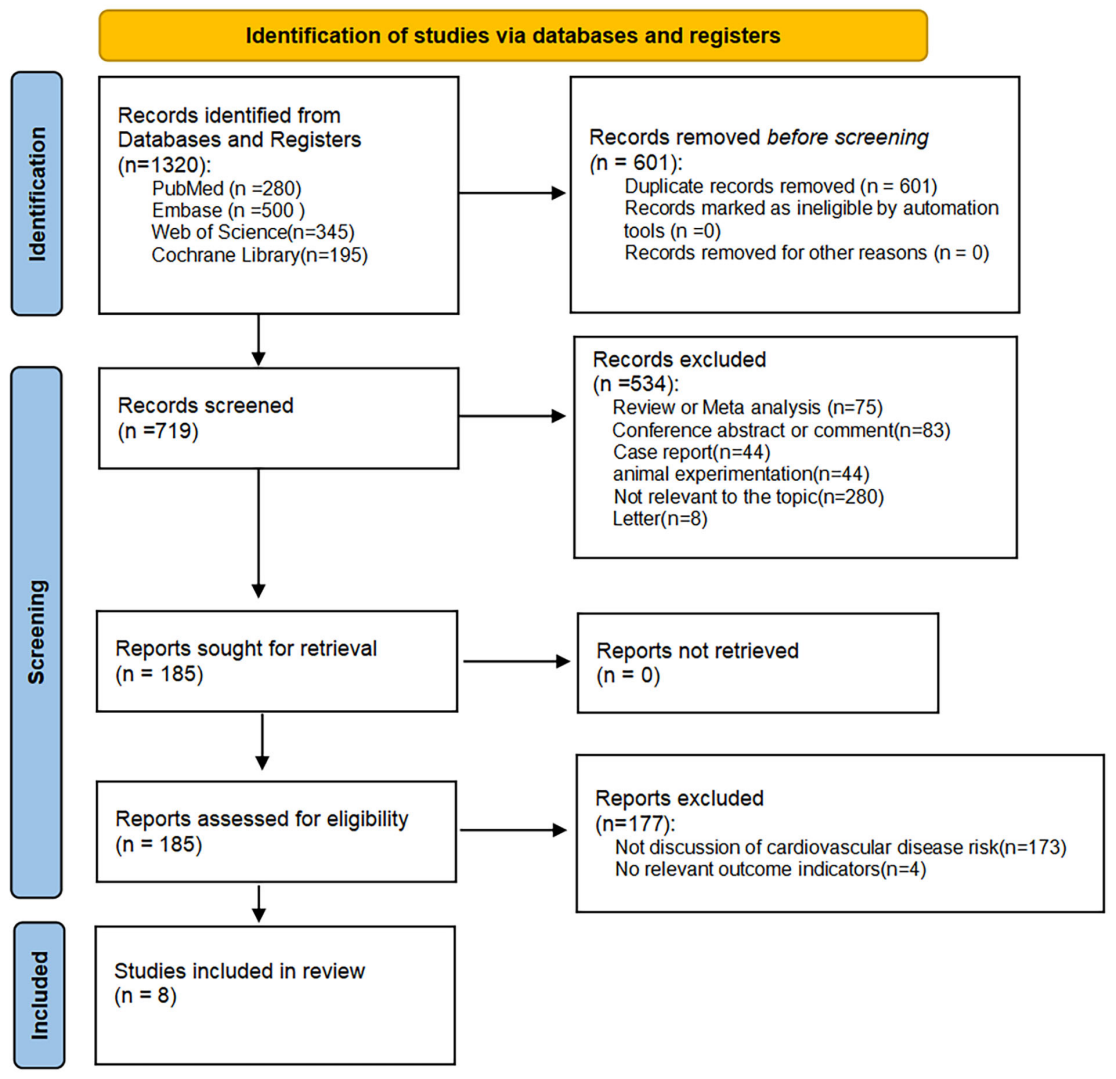
PRISMA flow diagram of study identification and inclusion process.

We obtained 1320 articles by searching multiple databases; 601 articles were excluded because of duplication, and 534 articles were excluded for the following reasons after reading the titles and abstracts: irrelevance to the topic of our study, systematic review, meta-analysis, conference abstracts, case reports, letters, and animal studies. Of the remaining 185 articles, 177 were excluded because they did not focus on cardiovascular disease risk and did not have relevant outcome indicators; thus, eight articles were included in our meta-analysis (23, 26–32) (**Figure 1**).

### 3.1 Study characterization and quality assessment

We included eight studies from five countries, including 138–065 patients (23, 26–32). The articles were published between 2021 and 2023: three from the US, two from China, and three from Italy, the United Kingdom, and Canada. One of these was a randomized controlled study and the remaining seven were retrospective cohort studies. Cardiovascular disease risks of interest for inclusion in the study included MACEs, stroke, all-cause mortality, myocardial infarction, heart failure, arrhythmia, and ischemic heart disease. We used the RoB 2 to assess the quality of the randomized controlled trial (29), which assessed some risk for both the randomization process and deviation from the established intervention components. This was due to differences in the mode of administration (subcutaneous versus [vs.] intramuscular) and frequency of administration (monthly vs. every 3 months) between degarelix and leuprorelin during the trial, which made it impossible to blind the patients and nurses who administered the drugs. For non-randomized controlled trials (23, 26–28, 30–32), we assessed study quality using the NOS, which showed that all studies scored between 7 and 9 and were of high quality. The characteristics of every included study are shown in **Table 1**.

**Table 1:** Characteristics and quality scores of included studies.

Author	Year	Country	Study design	Sample size	Drugs	Groups	Quality assessment
Chen et al. (26)	2021	China	Retrospective cohort	1998	Leuprorelin, Goserelin, Buserelin, Triptorelin VS. Degarelix	GnRH agonist 1332 Degarelix 666	9
Cicione et al. (27)	2023	Italy	Retrospective cohort	94030	Leuprorelin, Goserelin, Buserelin, Triptorelin VS. Degarelix	GnRH agonist 88902 Degarelix 5128	7
Davey et al. (23)	2021	UK	Retrospective cohort	9081	Leuprorelin, Goserelin, , Triptorelin VS. Degarelix	GnRH agonist 8980 Degarelix 101	7
Dragomir et al. (28)	2023	Canada	Retrospective cohort	10785	GnRH agonist VS. Degarelix	GnRH agonist 10201 Degarelix 584	8
Lopes et al. (29)	2021	USA	RCT	545	Leuprorelin VS. Degarelix	GnRH agonist 269 Degarelix 276	Some concerns
Merola et al. (30)	2022	USA	Retrospective cohort	3774	Leuprorelin VS. Degarelix	GnRH agonist 1887 Degarelix 1887	8
Shao et al. (31)	2023	China	Retrospective cohort	15626	Leuprorelin, Goserelin, Triptorelin VS. Degarelix	GnRH agonist 15127 Degarelix 499	9
Wallach et al. (32)	2021	USA	Retrospective cohort	2226	Leuprorelin VS. Degarelix	GnRH agonist 1113 Degarelix 1113	8

RCT, Randomized controlled trial.

### 3.2 Synthesis of results

**Figure 2:**
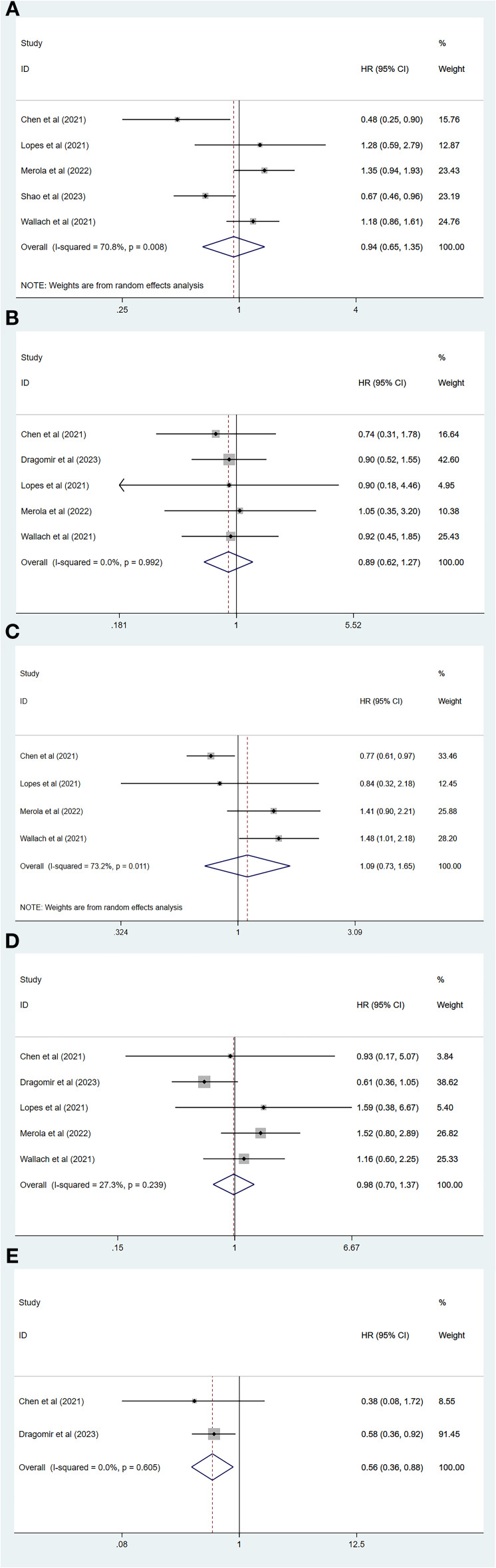
Forest plot comparing cardiovascular disease risk between degarelix and gonadotropin-releasing hormone agonists. **(A)** Forest plot comparing risk of major adverse cardiovascular event between degarelix and GnRH agonists. **(B)** Forest plot comparing risk of stroke between degarelix and GnRH agonists. **(C)** Forest plot comparing risk of all-cause mortality between degarelix and GnRH agonists. **(D)** Forest plot comparing risk of myocardial infarction between degarelix and GnRH agonists. **(E)** Forest plot comparing risk of heart failure between degarelix and GnRH agonists. HR, hazard ratio; CI, confidence interval.

**Figure 3:**
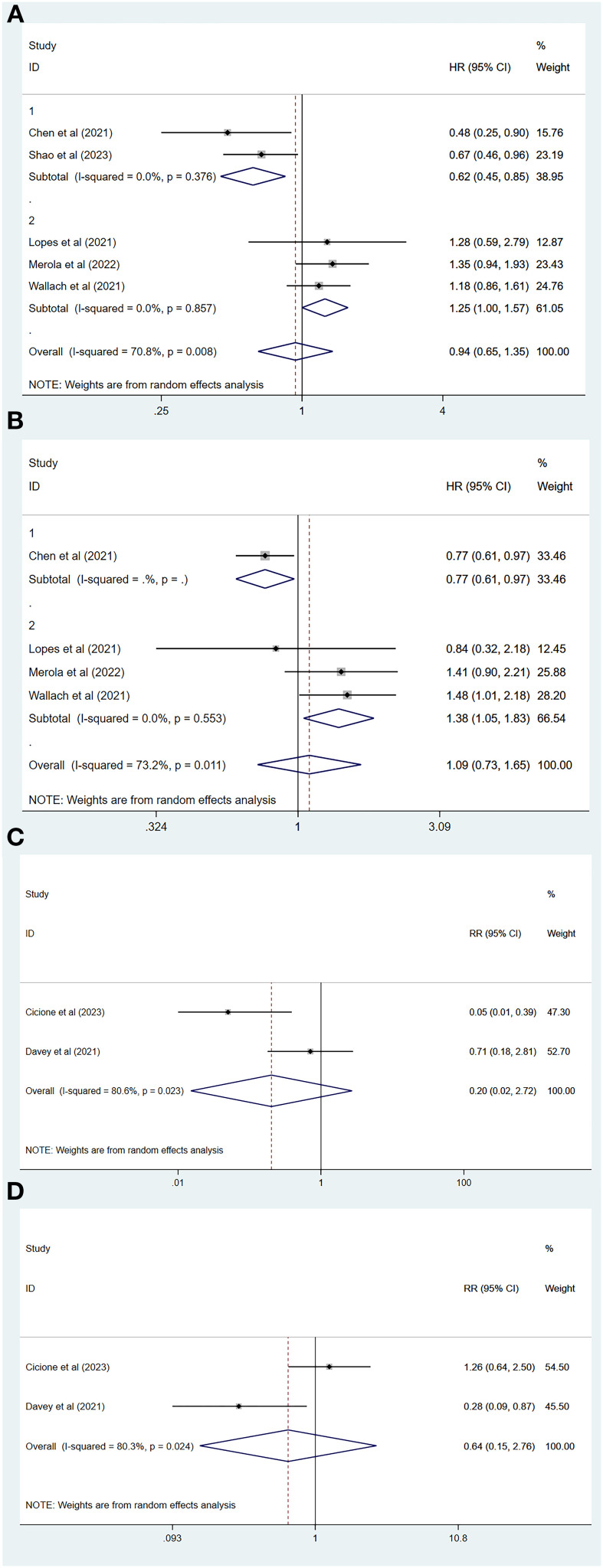
Subgroup analysis and forest plot with RR as a summary indicator. **(A)** Subgroup analysis comparing major adverse cardiovascular event between degarelix and GnRH agonists. **(B)** Subgroup analysis comparing all-cause mortality between degarelix and GnRH agonists. **(C)** Forest plot comparing risk of myocardial infarction between degarelix and GnRH agonists. **(D)** Forest plot comparing risk of arrhythmia between degarelix and GnRH agonists. RR, relative risk; CI, confidence interval.

Of all the included studies, five of which had MACEs as the endpoint (26, 29–32), our pooled results showed that the risk of MACEs was similar for both degarelix and GnRH agonists compared to each other (HR=0.94, 95% CI: 0.65–1.35; P=0.73). Because there was heterogeneity across studies (I^2=^70.8%, P=0.01, **Figure 2A**), a random-effects model was used, and a subgroup analysis was conducted to identify sources of heterogeneity. Of the five included studies, two compared degarelix with leuprorelin, triptorelin, goserelin, and buserelin (26, 31), and three compared degarelix with leuprorelin (29, 30, 32). We categorized the former into subgroup 1 and the latter into subgroup 2. The results suggested no heterogeneity within the two subgroups (subgroup 1: I^2=^0.0%, P=0.38; subgroup 2: I^2=^0.0%, P=0.86; **Figure 3A**); therefore, the difference in the contrasting drugs was considered a source of heterogeneity.

Five of all studies focused on stroke as the endpoint (26, 28–30, 32), and our combined results showed no significant difference in the risk of stroke between degarelix and GnRH agonists (HR=0.89, 95% CI: 0.62–1.27, P=0.52). A fixed-effects model was used because there was no heterogeneity among the five studies (I^2^ = 0.0%, P=0.99, **Figure 2B**).

A total of four studies had an endpoint of all-cause mortality (26, 29, 30, 32), and the pooled results suggested a similar risk of all-cause mortality between degarelix and GnRH agonists (HR=1.09, 95% CI: 0.73–1.65, P=0.67). We used a random-effects model to pool the results because of the significant heterogeneity among the studies (I^2=^73.2%, P=0.01, **Figure 2C**). Three of these studies compared degarelix to leuprorelin (29, 30, 32), and were included in a subgroup, with pooled results suggesting no heterogeneity among studies within this subgroup (I^2=^0.0%, P=0.55; **Figure 3B**). Therefore, the consideration of heterogeneity came from comparing degarelix with different GnRH agonists.

Five studies focused on myocardial infarction as the endpoint (26, 28–30, 32) and the combined results suggested that degarelix did not show a lower risk of myocardial infarction than GnRH agonists (HR=0.98, 95% CI: 0.70–1.37, P=0.91). Heterogeneity between the studies was not significant (I^2=^27.3%, P=0.24); therefore, a fixed-effects model was used (**Figure 2D**).

Two of all the articles focused on heart failure as a study endpoint (26, 28), and the combined results suggested that degarelix reduces the risk of heart failure (HR=0.56, 95% CI: 0.36–0.88, P=0.01). There was no heterogeneity among the studies (I^2=^0.0%, P=0.61); therefore, a fixed-effects model was used (**Figure 2E**). Because of the small number of included studies, publication bias and sensitivity analysis were not performed.

Two studies used RR as the outcome metric (23, 27), with the common endpoints of interest being myocardial infarction and arrhythmia, and the pooled results suggesting that degarelix and GnRH agonists have a similar risk of myocardial infarction (RR=0.20, 95% CI: 0.02–2.72, P=0.23; **Figure 3C**) and arrhythmia (RR=0.64, 95% CI: 0.15–2.76, P=0.55; **Figure 3D**). We combined the data using a random-effects model because of the heterogeneity between the two studies regarding myocardial infarction (I^2=^80.6%, P=0.02) and the two studies concerning arrhythmia (I^2=^80.3%, P=0.02). As there were not enough included studies, subgroup analysis, sensitivity analysis, and publication bias evaluations could not be performed.

### 3.3 Publication bias

**Figure 4:**
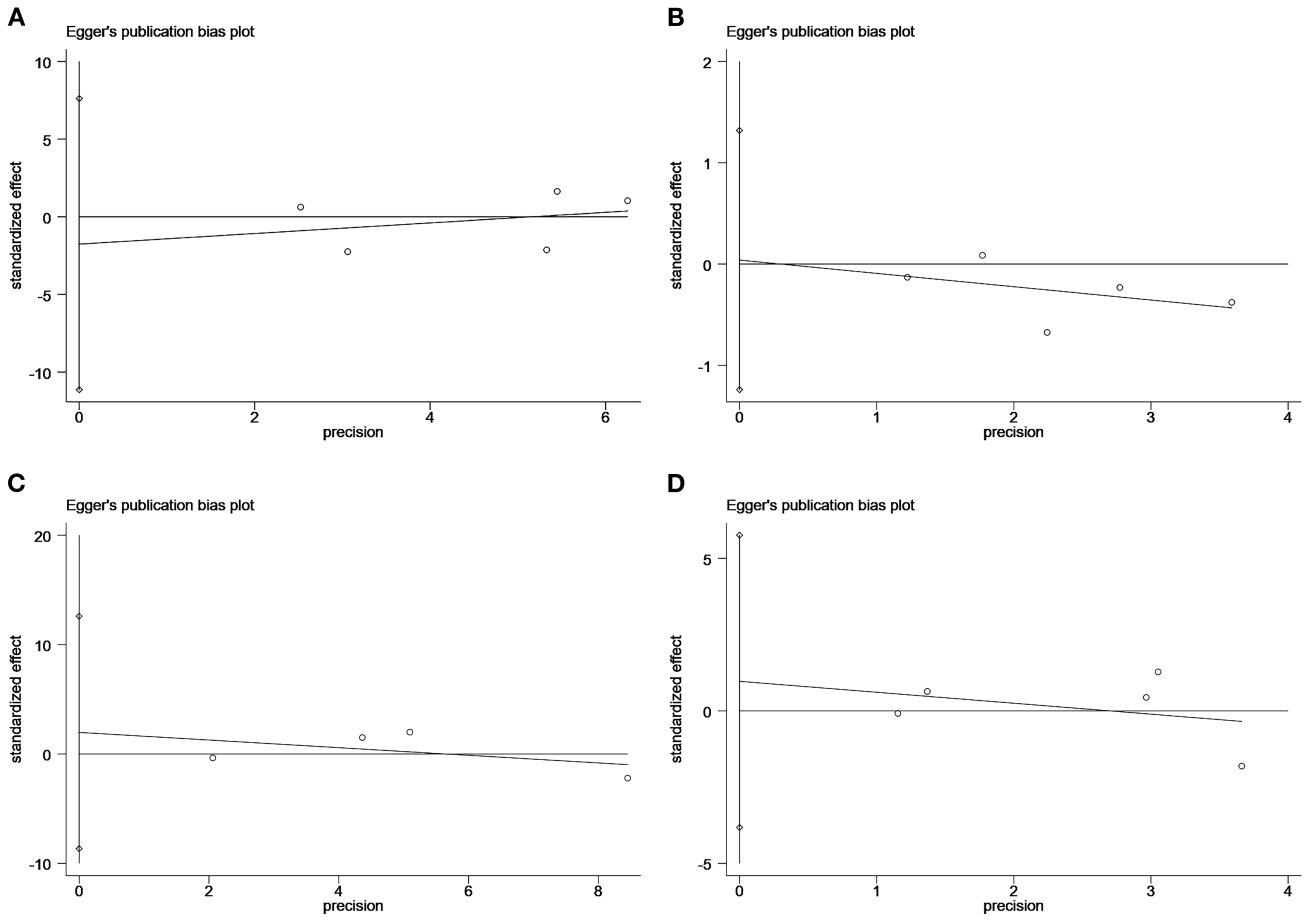
Plot of the Egger’s test for publication bias: major adverse cardiovascular event **(A)**; stroke **(B)**; all-cause mortality **(C)**; myocardial infarction **(D)**.

The Egger test showed no significant publication bias in studies with the following endpoints: MACEs (P=0.59), stroke (P=0.93), all-cause mortality (P=0.51), and myocardial infarction (P=0.57) (**Figure 4**).

### 3.4 Sensitivity analysis

**Figure 5:**
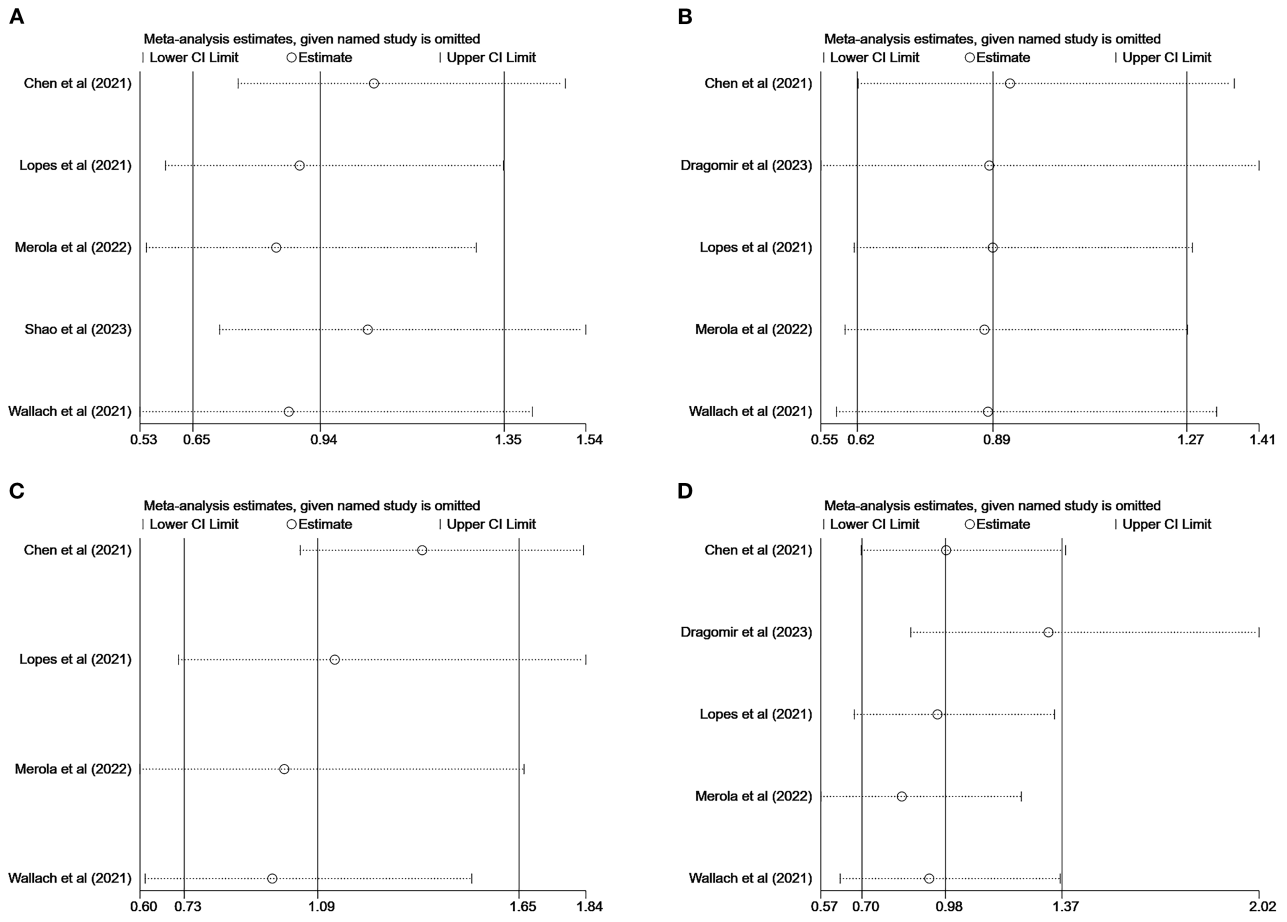
Sensitivity analysis: major adverse cardiovascular event **(A)**; stroke **(B)**; all-cause mortality **(C)**; myocardial infarction **(D)**.

We performed sensitivity analyses of articles with MACEs, stroke, all-cause mortality, and myocardial infarction as the endpoints using the literature-by-exclusion method. We found that the exclusion of any of the studies had no effect on the pooled results (**Figure 5**), suggesting that our results are reliable and robust.

## 4 Discussion

Comparing the risk of cardiovascular disease between degarelix and GnRH agonists was our main study objective, and the meta-analysis of the included studies suggested that there was no difference in the risk of MACEs, stroke, myocardial infarction, all-cause mortality, or arrhythmia between degarelix and GnRH agonists; however, degarelix was shown to reduce the risk of heart failure. These results are similar to those of a prospective international randomized clinical trial (PRONOUNCE trial) (29).

There are currently conflicting views regarding whether ADT in patients with prostate cancer increases the risk of cardiovascular disease. A pooled analysis of the results of eight randomized trials by Nguyen et al. (33) showed that the risk of cardiovascular death was similar in patients who received ADT compared to controls (RR=0.93, 95% CI: 0.79–1.10, P=0.41). Similarly, an opinion by Alibhai et al. (34) suggests that the continuous use of ADT for at least 6 months is linked to a higher risk of diabetes mellitus (HR=1.16, 95% CI: 1.11–1.21) and fragility fracture (HR=1.65, 95% CI: 1.53–1.77), but there is no increased risk of sudden cardiac death (HR=0.96, 95% CI: 0.83–1.10) or acute myocardial infarction (HR=0.91, 95% CI: 0.84–1.00). However, Cardwell et al. (7) reported that ADT leads to a 30% increased risk of cardiovascular events (HR=1.30, 95% CI: 1.20–1.40) and suggested that both GnRH agonists (HR=1.30, 95% CI: 1.20–1.40) and degarelix (HR=1.50, 95% CI: 1.20–1.90) lead to an increased risk of cardiovascular events. In addition, Taylor et al. (8) reported a 17% increase in cardiovascular-related mortality with the use of ADT in patients with prostate cancer (HR=1.17, 95% CI: 1.07–1.29).

GnRH agonists and antagonists induce and maintain testosterone suppression, and there is a positive correlation between physiologic testosterone levels and vascular health; low testosterone levels are associated with hypertension, decreased bone density, abnormal glucose metabolism, and increased cardiovascular risk (35, 36). These adverse effects are part of metabolic syndrome. Muller et al. (15) conducted a cross-sectional study and found that higher testosterone levels in men were independently associated with increased insulin sensitivity and reduced risk of metabolic syndrome. Similarly, a longitudinal study by Laaksonen et al. (16) showed that low testosterone levels in men led to an increased risk of metabolic syndrome and diabetes mellitus. The use of ADT in patients with prostate cancer leads to a higher percentage of abdominal obesity and a higher prevalence of hyperglycemia, which may lead to increased body mass index, dyslipidemia, and decreased insulin sensitivity (37–39). Men with metabolic syndrome have an increased risk of cardiovascular disease and all-cause mortality even in the absence of baseline cardiovascular disease or diabetes mellitus (40, 41). Although GnRH agonists and antagonists have different mechanisms of action, they both suppress testosterone, which may explain the similarity in cardiovascular disease risk between the two.

Some studies have suggested that GnRH agonists are associated with a higher risk of cardiovascular disease than antagonists, possibly because of the differences in FSH levels between the two. GnRH agonists activate the expression of GnRH receptors in pituitary cells, leading to elevated FSH levels, which begin to decrease when GnRH receptors in pituitary cells are gradually desensitized (5), whereas GnRH antagonists directly inhibit FSH and LH production by rapidly and competitively binding to the GnRH receptor and blocking GnRH from binding to its receptor. FSH levels in patients treated with GnRH agonists do not fall as low as those in patients treated with GnRH antagonists because the former primarily inhibit LH, whereas the latter inhibit both LH and FSH (14). Based on the differences in FSH levels, some researchers have hypothesized that FSH affects cardiovascular diseases. The results of an animal study by Han et al. (5) suggested that FSH leads to the progression of atherosclerosis and destabilizes plaques by promoting the inflammatory response and migration of macrophages. Similarly, Wang et al. (14) reported that FSH accelerates atherosclerosis by exacerbating endothelial inflammation and promoting endothelial adhesion of monocytes, thereby contributing to ADT-associated cardiovascular disease. We speculate that degarelix’s reduction of the risk of heart failure may be related to the following mechanisms: Firstly, as a GnRH antagonist, degarelix can rapidly and directly lower testosterone levels, which may reduce the direct adverse effects of androgens on the heart. Secondly, degarelix may improve cardiovascular function by regulating inflammatory responses and enhancing endothelial function. Moreover, the mechanism and hormonal level changes of degarelix differ from those of GnRH agonists, which may be the reason for the differences in cardiovascular endpoint risks. For example, GnRH agonists have a “flare-up” phenomenon, which may have adverse effects on the cardiovascular system.

We compared the risk of cardiovascular disease between degarelix and GnRH agonists by performing a systematic and comprehensive search of databases, and subgroup and sensitivity analyses demonstrated the reliability and stability of the results. The results of this study may have certain significance for clinical treatment decisions: First, in terms of risk assessment, a comprehensive cardiovascular risk assessment was conducted for all prostate cancer patients, including medical history, physical examination and necessary laboratory tests; Secondly, in terms of treatment options, for patients with a history of cardiovascular diseases or a high risk of cardiovascular events, digarec may be a better choice. Thirdly, in terms of risk management, all prostate cancer patients receiving ADT should receive active cardiovascular risk management, including lifestyle intervention and drug treatment. Closely monitor the cardiovascular conditions of patients receiving degarix treatment.

In addition, this article also has potential utility in other fields: First, in oncology and endocrine therapy, the methods of this study can be extended to the drug safety assessment of other hormone-dependent cancers (such as breast cancer), and compare the cardiovascular risks of different endocrine therapies; Secondly, in terms of cardiovascular drug safety research, similar methods can be used to evaluate the cardiovascular effects of new hypoglycemic drugs or immune checkpoint inhibitors; Thirdly, in terms of drug regulation and clinical guideline formulation, regulatory agencies (such as the FDA and EMA) can refer to such meta-analyses to optimize drug safety warnings or indication recommendations, and clinical guidelines (such as NCCN and ESC) can adjust treatment recommendations based on high-quality evidence, such as giving priority to drugs with lower cardiovascular risks. Fourth, in terms of integrating real-world evidence (RWE), in the future, randomized controlled trials (RCTS) and real-world data (such as electronic health records) can be combined to further verify the conclusions of meta-analyses. However, there are some limitations to our study. Among the included studies, only one was a randomized controlled trial (RCT), and the remaining seven were retrospective cohort studies. Retrospective studies are vulnerable to selection bias, information bias and confounding factors (for example, factors such as patients’ baseline cardiovascular risk, comorbidities, lifestyle, etc. may affect the research results), which may affect the reliability of the research results. The evidence level of RCT is higher, but this study has some risks in terms of deviations in the randomization process and intervention measures, which may affect the interpretation of the research results. At present, the RCT studies for diagnosing Degarelix are limited and a sufficient number have not been included in this article.

## 5 Conclusion

Overall, the risks of MACEs, stroke, myocardial infarction, all-cause mortality, and arrhythmia were similar between degarelix and GnRH agonists; however, degarelix reduced the risk of heart failure. There is a need to monitor the potential side effects of ADT, especially in patients with cardiovascular disease at baseline. Regarding the effects of different ADT modalities on cardiovascular disease, larger prospective randomized controlled trials are needed for further clarification.
